# The relationship between changes in peak expiratory flow and asthma exacerbations in asthmatic children

**DOI:** 10.1186/s12887-024-04754-7

**Published:** 2024-04-27

**Authors:** Xiongbin Chen, Peng Han, Yan Kong, Kunling Shen

**Affiliations:** 1 Respiratory Department, Beijing Children’s Hospital, Capital Medical University, China National Clinical Research Center of Respiratory Diseases, National Center for Children’s Health, Beijing, China 100045; 2 Department of Respiratory, Shenzhen Children′s Hospital, 518038 Shenzhen, China

**Keywords:** Peak expiratory flow, Asthmatic children, China children’s asthma action plan

## Abstract

**Background:**

Asthma is one of the most common chronic airway diseases in children. Preventing asthma exacerbation is one of the objectives of all asthma action plans. In patients with poor perception, it is difficult to identify acute asthma exacerbations by clinical asthma score, asthma control test or asthma control questionnaire. The aim of this study is to analyze whether children with asthma have changes in peak expiratory flow(PEF)before an acute asthma exacerbation and to evaluate the relationship between PEF and asthma exacerbation.

**Methods:**

Basic information (including sex, age, atopy, etc.) and clinical information of asthmatic children who registered in the Electronic China Children’s Asthma Action Plan (e-CCAAP) from 1 September 2017 to 31 August 2021 were collected. Subjects with 14 consecutive days of PEF measurements were eligible. Subjects in this study were divided into an exacerbation group and a control group. We analyzed the relationship between changes in PEF% pred and the presence of asthma symptoms.

**Result:**

A total of 194 children with asthma who met the inclusion criteria were included, including 144 males (74.2%) and 50 females (25.8%), with a male-to-female ratio of 2.88:1. The mean age of the subjects was 9.51 ± 2.5 years. There were no significant differences in sex, age, allergy history or baseline PEF between the two groups. In children with and without a history of allergy, there was no significant difference between the variation in PEF at 14 days. Patients who only had a reduced in PEF but no symptoms of asthma exacerbation had the greatest reduction in PEF compared to the other groups. The most common cause of acute exacerbations of asthma is upper respiratory tract infection. Among the causes of acute exacerbations of asthma, the variation in PEF caused by air pollution was significantly higher than that of other causes (*P* < 0.05). In acute exacerbations, the decrease in PEF was significantly greater in the exacerbation group than in the control group. In children with asthma symptoms, there was a decrease in PEF approximately 1.34 days before the onset of symptoms.

**Conclusion:**

Children with asthma show a decrease in PEF 1.34 days before the onset of asthma symptoms. We recommend that asthmatic children who show a decrease in PEF should step-up asthma therapy. The most common cause of acute exacerbations of asthma was upper respiratory tract infections, and the variation in PEF caused by air pollution was significantly higher than that caused by other factors.

## Background

In many countries, childhood asthma is a major public health problem [1]. As of 2018, there were approximately 350 million people with asthma worldwide (including children, adolescents and adults) [2]. The Third National Epidemiological Survey of Childhood Asthma in China found that the prevalence of asthma among urban children aged 0–14 years was 3.02% in 2010, compared with 1.09% and 1.97% in 1990 and 2000, respectively [3]. In China, the incidence of childhood asthma is increasing at a rate of more than 50% per decade. According to the national report in China, the overall prevalence of asthma in adults is 4.2% (95% CI 3.1–5.6), representing 45.7 million people [4].

A global multicenter study showed high rates of poorly controlled asthma in children (6–7 years), adolescents (13–14 years), and adults (≥ 19 years), with particularly high rates in children [5]. The Global Initiative for Asthma (GINA) states that the key to asthma management is the prevention of acute exacerbations and that early identification of asthma exacerbations, and timely intervention can reduce the burden of disease [6]. However, asthma exacerbations usually occur without any signs, and many children with asthma can breathe normally for weeks or months between exacerbations. Individuals and families do not have an accurate perception of symptoms. We need objective parameters to describe the severity of asthma. Peak expiratory flow (PEF) is an objective pulmonary parameter measured by an instrument that gives a true picture of a child’s airways. GINA recommends PEF testing and regular follow-up for children over the age of 5 with asthma prior to diagnosis and initiation of controller therapy [6].

It had been shown that patients’ perceptions of asthma symptoms or the severity of exacerbations vary and that difficulties with physical sensations and emotional expression were often associated with severe asthma, even fatal acute asthma attacks [7]. In children, the need for additional objective parameters to describe the status and severity of asthma is reinforced by the poor perception of asthma symptoms and the fact that children often have difficulty expressing themselves. Studies have shown that forced expiratory volume in one second (FEV_1_) does not change significantly in most school-aged children, regardless of whether they have an acute asthma attack, and FEV_1_ is not associated with asthma severity as defined by symptoms [8–10]. A study in adults suggests that PEF may be a useful method for monitoring trends in asthma exacerbations and quantifying asthma control history [11]. Several studies have shown the effectiveness of PEF-based asthma education and self-management programs in reducing emergency department visits and hospitalizations due to asthma exacerbations [12–14]. Compared with FEV1, PEF can be self-tested at home, making it more feasible and easier to comply with. Numerous guidelines suggest that PEF is a valuable, readily available measure that is well suited to monitoring long-term trends in asthma control [6, 15]. The PEF test helps to identify the variable nature of airflow limitation (obstruction) in patients, which is a central feature of asthma.

Many international guidelines recommend the provision of a written asthma action plan (WAAP) to guide patients in recognizing and responding to worsening asthma symptoms to reduce acute asthma exacerbations [6, 16, 17]. Almost all written asthma action plans (WAAPs) include long-term monitoring of PEF as an objective indicator of asthma control.

However, previous studies were mostly conducted in professional medical institutions under the guidance of medical professionals. It is difficult to reflect the management of asthma in family conditions. And most of the studies were limited to adults, with few studies in children.

To help healthcare professionals, children and families self-manage asthma and achieve good asthma control, we implemented the China Children’s Asthma Action Plan (CCAAP) and published an expert consensus on the clinical application of the CCAAP (in Chinese with an English abstract) [18].

This study was based on the CCAAP. We collected the children’s PEF values and combined them with the children’s symptoms to determine their asthma status. The aim of this study is to show the relationship between changes in PEF and asthma exacerbations to help children better manage their asthma.

## Methods

### Inclusion criteria

1. Registered in the electronic China Children’s Asthma Action Plan (e-CCAAP).
2. Age ≥ 6 and ≤ 18 years.
3. Children diagnosed with asthma by physicians [19].
4. The PEF measurements were recorded for at least 14 consecutive days.

### Exclusion criteria

1. PEF values with significant deviations.
2. The information is incorrect.

We checked the children’s medical records to make sure the information was correct. For children who did not have a medical record, we would contact their parents or guardians to verify that the information was correct. If their parents or guardians could not be contacted, the information would be considered incorrect. And We defined significant deviations as PEF values that were significantly lower (e.g., below 10 L/min) or significantly higher than the baseline PEF(e.g., greater than 13,245 L/min).

### Subjects

Asthmatic children who registered in the e-CCAAP from 1 September 2017 to 31 August 2021 and recorded PEF measurements for at least 14 consecutive days were eligible. According to CCAAP, children were required to step up treatment when their PEF%≤80% and/or onset of asthma symptoms.

Patients with asthma exacerbations had to increase the dose of inhaled corticosteroids (ICS) by 2 to 4 times, which depending on the dose before the asthma exacerbation and the severity of the asthma and giving Salbutamol at the same time. If patients had any of the following symptoms, they needed to visit doctors:1) Heavy breathing, suffocation, dyspnea, crying, irritability, etc;2) Initial treatment with inhaled bronchodilators was ineffective or situations getting worsen. And they also can used ICS-LABA(Long-acting β-agonists) composite formulation.It was a case control study. We retrospectively analyzed their acute asthma exacerbations and PEF% pred. Basic information included sex, age, allergy history, and basic medications. History of allergy was defined as at least 1 positive for inhaled allergen (dust mites, mold, cockroaches, pets, spring/fall pollen, other allergens) or at least 1 positive for food allergen (milk, egg, wheat, nuts, seafood, soybeans, peanuts, other allergens).In our study, PEF reduction was defined as PEF% pred ≤ 80%. Subjects were divided into an exacerbation group or a control group according to asthma symptoms and PEF% pred. The characteristics of the control group were PEF% pred > 80% and no onset of asthma symptoms. The participants were asked to take PEF measurements three times in the morning and three times in the evening and to record the optimum value of the measurement. The triggers of asthma exacerbations were recorded in e-CCAAP. The exacerbation group were divided into three subgroups: (1) onset of asthma symptoms after a decrease in PEF (PEF + symptom group). (2) only a decrease in PEF (PEF group). (3) onset of asthma symptoms only (symptom group).

### Definition of air pollution and climate change

In this study, the definition of air pollution was Air Quality Index (AQI)>100 [20]. The AQI is provided by the Ministry of Environmental Protection of China. The AQI is available in real time for most cities on the website and smartphone app. Climate change is mainly about colder weather, which leads to the inhalation of cold air.

### Analysis

Statistical analyses were performed using SPSS Statistics version 22.0. Continuous and categorical variables were presented as the mean ± standard deviation (‾×±sd) or number (percentage). We analyzed the differences in the demographic characteristics between the two groups by using a χ^2^ test and two-sample t tests for proportions and continuous data, respectively. Comparisons of nonnormally distributed data were performed using the Mann‒Whitney U rank test. A two-tailed p value of 0.05 was considered to be statistically significant.

### Ethics approval and consent to participate

The study was approved by the Ethics Committee of Beijing Children’s Hospital, and written informed consent was waived.

## Result

### Baseline characteristics

The baseline characteristics of the two groups were summarized in Table 1. A total of 194 subjects were included in this study. Of the 194 subjects, there were 144 males and 50 females, with a male to female ratio of 2.88:1. The mean age was 9.51 ± 2.5 years. There were 162 subjects in the exacerbation group. Of the 162 subjects, 98 were in the PEF + symptom group, 13 in the PEF group and 51 in the symptom group. There were 32 subjects in the control group. The number of subjects in the PEF + symptom group was significantly higher than that in the other groups (*P*<0.05). The mean ages of the four groups were 9.56 ± 2.08, 10.69 ± 2.39, 9.41 ± 3.03, and 9 ± 2.78 years, respectively. The baseline PEF was significantly higher in the PEF group than in the other three groups (*P* = 0.014). With the exception of baseline PEF, there were no statistically significant differences in baseline characteristics between the two groups. Most subjects were from eastern China. (Table 1).

**Table 1:** Baseline characteristics

	Exacerbation group(*n* = 162)			Control group(*n* = 32)	P
Characteristic	PEF + symptom group(*n* = 98)	PEF group(*n* = 13)	Symptom group(*n* = 51)		
**Gender**					0.141
male	77(78.6%)	11(84.6%)	37(72.5%)	19(59.3%)	
female	21(21.4%)	2(15.4%)	14(27.5%)	13(40.7)	
**Age, mean ± SD, y**	9.56 ± 2.08	10.69 ± 2.39	9.41 ± 3.03	9 ± 2.78	0.228
6–9	49(50%)	4(30.8%)	35(68.6%)	16(50%)	
10–14	41(41.8%)	6(46.2%)	13(25.5%)	4(12.5%)	
15–18	8(8.2%)	3(23.1%)	3(5.9%)	12(37.5%)	
**Allergy history**					0.698
Yes	64(65.3%)	10(76.9%)	38(74.5%)	22(68.8%)	
No	34(34.7%)	3(23.1%)	13(25.5%)	10(31.2%)	
**Resident Region**					0.0001
**East China**	25(25.5%)	10(76.9%)	37(72.5%)	26(81.3%)	
**West China**	71(72.4%)	1(7.7%)	10(19.6%)	0	
**Central China**	2(2.1%)	2(15.4%)	4(7.9%)	6(18.7%)	
**Baseline PEF(l/min, ±2SD)**	237.73 ± 75.93	270.08 ± 83.04	239.53 ± 100.69	215.31 ± 87.64	0.014*
**Baseline Medication**					0.366
LABA + ICS	69(70.4%)	8(61.5%)	37(72.5%)	20(62.5%)	
ICS	15(15.3%)	1(7.7%)	8(15.7%)	3(9.4%)	
others*	14(14.3%)	4(30.8%)	6(11.8%)	9(28.1%)	

LABA: Long Acting β 2 Agonist, ICS: inhaled corticosteroids. LABA + ICS include Budesonide and Formoterol Fumarate Powder for Inhalation, Salmeterol Xinafoate and Fluticasone Propionate. ICS includes Fluticasone, budesonide. Others includes Salbutamol

### Impact of allergy history on PEF

The distribution of food allergens or inhalation allergens in this study was shown in Table 2. There were no statistically significant differences between the groups for food and inhalation allergens. In children with a history of allergy, the mean PEF variation was less than that in children without a history of allergy, and their PEF decrease was less than that in children without a history of allergy when there was an onset of asthma symptoms, but the difference in PEF changes between these two groups was not statistically significant (*P* = 0.206).

**Table 2:** Distribution of allergens

	Exacerbation group(*n* = 162)			Control group(*n* = 32)	P
Characteristic	PEF + symptom group(*n* = 98)	PEF group(*n* = 13)	Symptom group(*n* = 51)		
**Food allergens, n(%)**					0.419
Soybeans	1(1.02%)	0(0.0)	0(0.0)	0(0.0)	
Seafood	1(1.02%)	0(0.0)	0(0.0)	0(0.0)	
Egg	5(5.1%)	0(0.0)	7(13.73%)	5(15.63%)	
Milk	8(8.16%)	3(23.08%)	7(13.73%)	1(3.13%)	
Wheat	1(1.02%)	0(0.0)	0(0.0)	0(0.0)	
Other allergens	82(83.68%)	10(76.92%)	37(86.27%)	26(81.24%)	
**Inhalation allergens, n(%)**					0.291
Dust mites	44(44.9%)	6(46.15%)	24(47.06%)	20(62.5%)	
Mold	6(6.12%)	2(15.38%)	3(5.88%)	0(0.0)	
Pets	1(1.02%)	0(0.0)	0(0.0)	0(0.0)	
Spring/Fall pollen	4(4.08%)	2(15.38%)	3(5.88%)	0(0.0)	
Cockroaches	1(1.02%)	2(15.38%)	0(0.0)	0(0.0)	
Other allergens	42(42.86%)	3(23.08%)	21(41.18%)	12(37.5%)	

### Changes in PEF over 14 days in included subjects

**Fig. 1:**
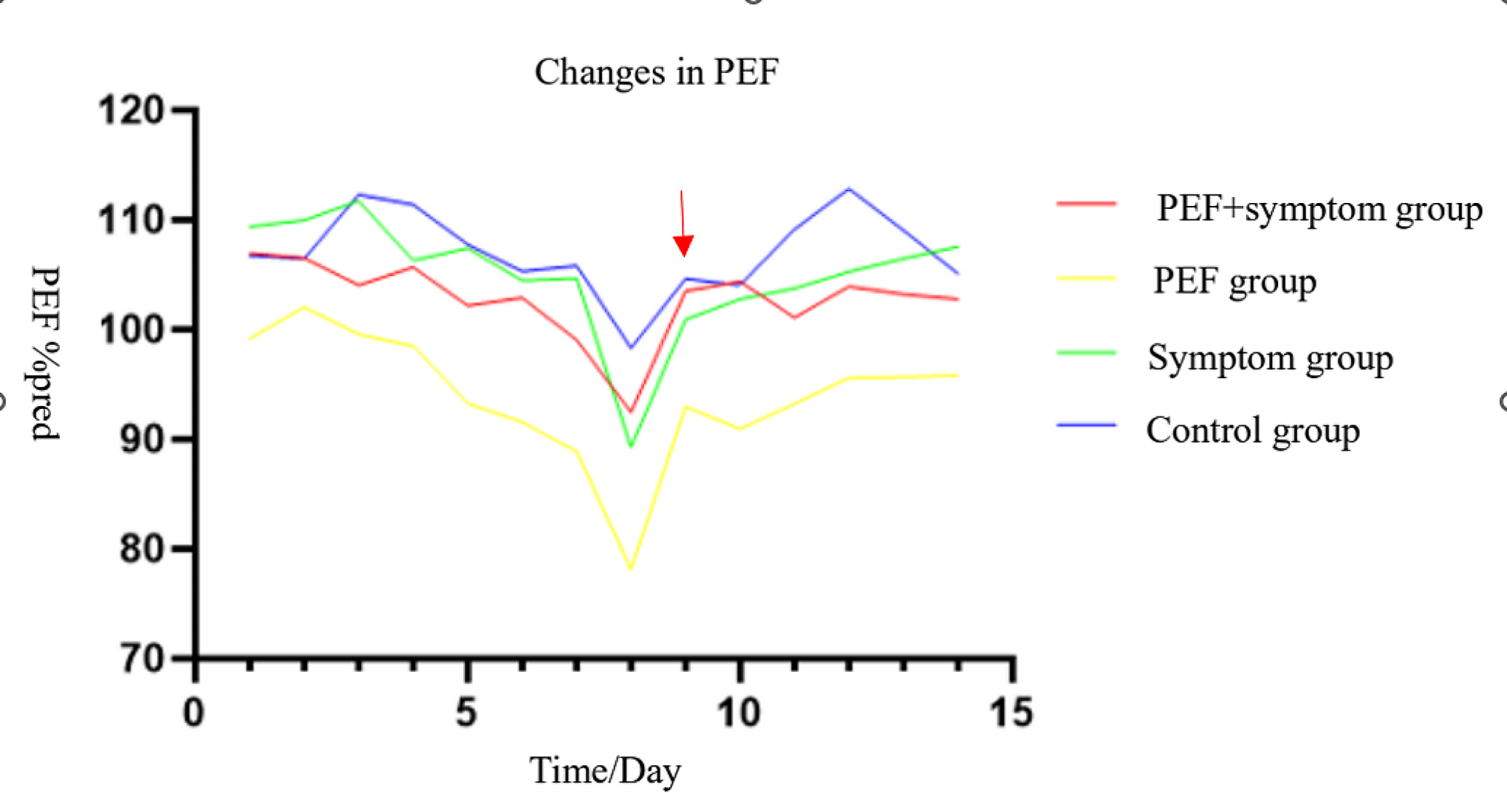
Changes in PEF. Changes in PEF in four groups in 14 days. The PEF group had greater changes. Red arrow indicates onset of asthma symptom

As shown in Fig. 1, the change in PEF% pred over 14 days in the exacerbation group (PEF + symptom group, PEF group, Symptom group) and the control group. The variation in PEF% pred was significantly greater in the PEF group than in the other groups.

### Analysis of triggers in the exacerbation group

**Fig. 2:**
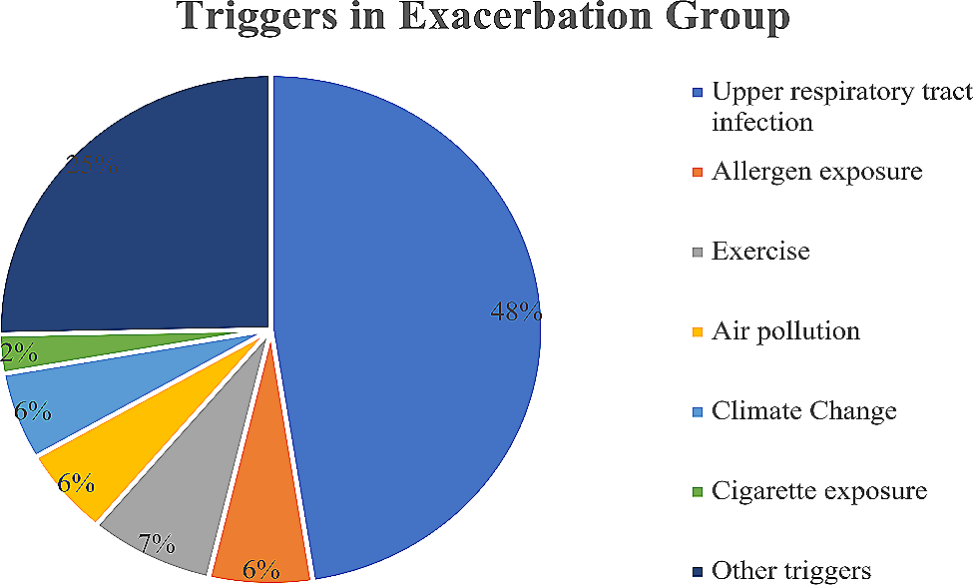
Triggers in the Exacerbation Group. Upper respiratory tract infection was the most common triggers

**Fig. 3:**
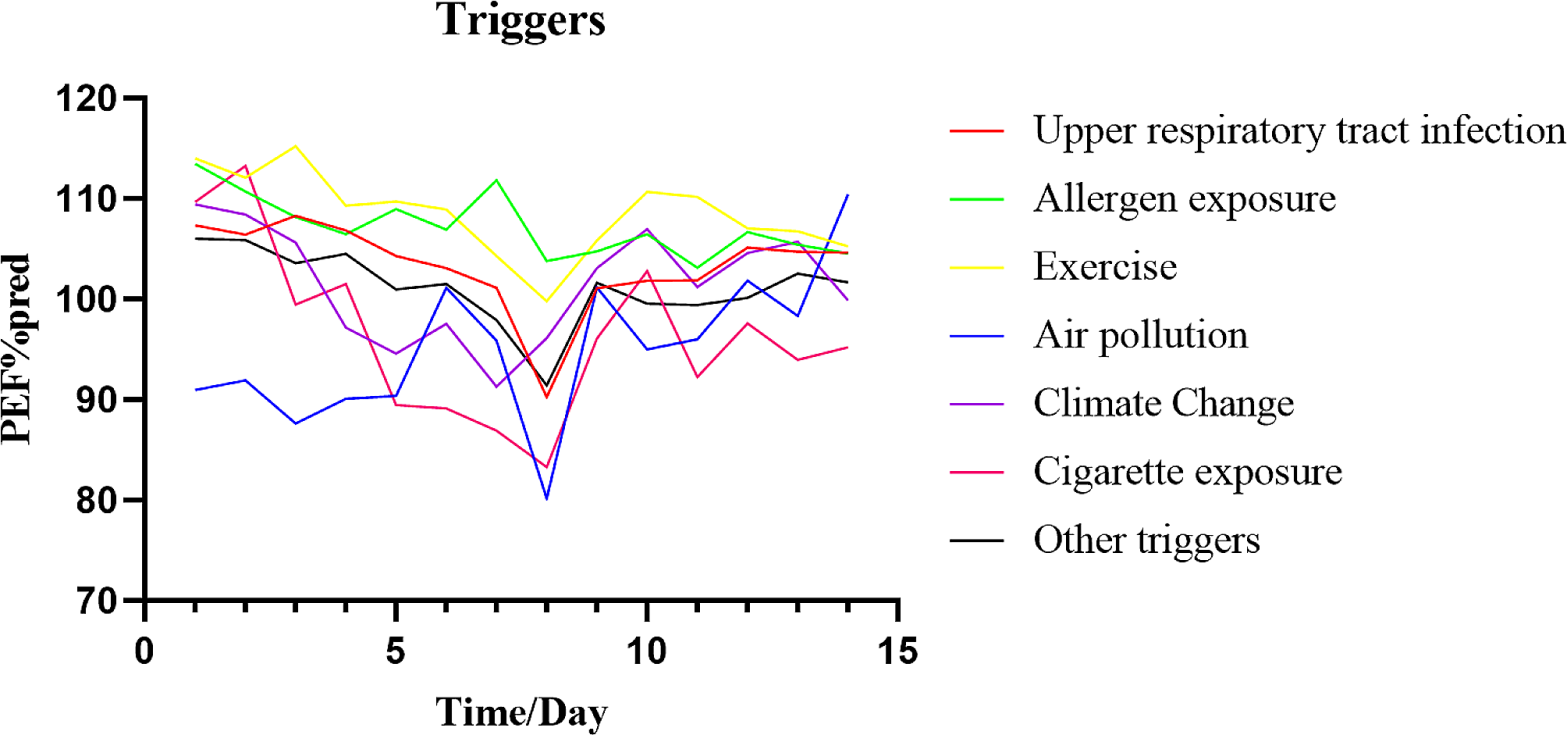
Changes in PEF with different triggers. Air pollution has significantly higher variations in PEF% pred than other triggers

As shown in Fig. 2 and Table 3, in the exacerbation group, the most common trigger for an acute exacerbation of asthma was upper respiratory tract infections. The proportion of acute exacerbations caused by upper respiratory tract infections in the PEF + symptom group, PEF group and symptom group was 44.9%, 61.5% and 49% respectively, which was significantly higher than that caused by other triggers (*P*<0.0001).Changes in PEF% pred for acute exacerbations of asthma caused by different triggers were shown in Fig. 3.

Acute exacerbations due to air pollution had significantly higher variations in PEF% pred than other triggers. Acute exacerbations of exercise-induced asthma had significantly less variating changes in PEF% pred than other triggers.

**Table 3:** Triggers in the Exacerbation Group

	Exacerbation group(*n* = 162)			P
	PEF + symptom group(*n* = 98)	PEF group(*n* = 13)	Symptom group(*n* = 51)	
**Triggers, n(%)**				
Upper respiratory tract infection	44(44.9%)	8(61.5%)	25(49.0%)	0.0001*
Allergen exposure	7(7.1%)	0(0.0)	3(5.9%)	0.206
Exercise	5(5.1%)	1(7.7%)	6(11.8%)	0.174
Air pollution	6(6.1%)	1(7.7%)	2(3.9%)	0.097
Climate change	8(8.2%)	0(0.0%)	1(2.0%)	0.02
Cigarette exposure	2(2.0%)	1(7.7%)	1(2.0%)	0.779
Other triggers*	26(26.6%)	2(15.4%)	13(25.4%)	0.0001

*Other triggers: included cry, laugh or pungent smell

### Time to PEF changes before exacerbation

We performed a retrospective analysis of children who developed asthma symptoms after the onset of PEF changes. Ninety-eight subjects (50.5%) developed symptoms after a decrease in PEF% pred, significantly more than those who did not develop asthma symptoms after a decrease in PEF% pred (6.7%). We found that most children with asthma had changes in PEF approximately 1–3 days before the onset of asthma symptoms. Statistically, the time from PEF change to symptom onset was 1.34 days [95% CI, 1.19, 1.49].

## Discussion

In our study, we found that children with asthma had a decrease in PEF 1.34 days before the onset of asthma symptoms, which may be an early sign of an acute exacerbation of asthma. A randomized controlled trial in children aged 7–14 years with moderate asthma showed that the PEF threshold was 70% of the optimum for increasing inhaled steroids and 50% of the optimum for starting prednisone therapy based on the PEF action plan. There was a significant decrease in PEF approximately 1 day prior to step-up treatment [21]. A study in adults showed that asthma exacerbations were characterized by a gradual decrease in PEF over a few days, followed by more rapid changes over 2 to 3 days [22]. Many countries’ asthma action plans advocated increasing the dose of inhaled corticosteroids (ICS) or initiating “yellow zone” therapy at the early signs of an exacerbation to avoid an acute exacerbation or reduce the severity and to prevent the need for oral steroids or hospitalization. In this study, some of the subjects had a significant decrease in PEF but had no asthma symptoms. This suggested that we may delay treatment if symptoms were used as an early sign of an acute exacerbation of asthma. PEF decreased before the onset of symptoms, and as soon as a decrease in PEF is detected during daily PEF monitoring, we can initiate “yellow zone” therapy. In our study, children in the PEF group had the greatest decrease in PEF% pred, which may be related to the fact that they had a higher PEF at baseline.

This study found that the most common trigger of acute asthma exacerbations in children was upper respiratory tract infections. Infection is the main trigger for acute exacerbations of asthma in children of all ages, followed by exposure to allergens [23]. In the United States, childhood asthma morbidity decreased during the novel coronavirus epidemic compared with other periods, which may be related to reduce pathogen exposure due to the use of masks [24]. The study by Anneclaire et al. also found that the likelihood of children’s asthma worsening increased as pollen levels increased [25]. Some studies found that houses that have been painted in the past 1 year are also a risk factor for acute exacerbations of asthma [26]. We need individualized action plans to improve the management of asthma, and avoiding infections and allergens are very important measures.

In this study, we found no significant differences in food allergens and inhalation allergens between the exacerbation group and the control group. Although allergen exposure is the second most common trigger. However, a study showed that, blood eosinophils and mold sensitization were significantly associated with asthma severity [27].The study by Zoratti, E. M. et al. also indicated that severe asthma often co-clusters with highly allergic children [28].More studies are needed to confirm the relationship between allergens and PEF.

Doctors, teachers and parents need to be involved in improving asthma control in children. Natasha et al. showed that asthmatic students, teachers, and family members were involved in the study together to teach them how to identify asthma symptoms based on the Asthma Action Plan (AAP) and actions for each area. By the end of the study, all students accurately identified symptoms, AAP areas, and action steps [29]. When we promote our asthma action plan, we can consider a combined hospital-school-home model.

The advantage of this study is the simplicity and economy of long-term PEF monitoring through an electronic platform, with subjects from all over China participating in this study. Subjects can monitor the PEF anytime and anywhere, which greatly improves their compliance. The limitations of this study are as follows. First, as a retrospective study, some recall bias was inevitable. Second, PEF measurements were related to the child’s ability to breathe calmly and regularly, measurements taken at home or at school were highly arbitrary, and even PEF measurements taken under different circumstances can vary greatly. Patients with upper respiratory tract infections may develop tonsillitis, which would cause upper airway obstruction and affect PEF measurement results. This would limit the use of PEF in daily life.

## Conclusion

In children with asthma, the PEF% pred decreased 1.34 days before the onset of asthma symptoms. Therefore, we recommend starting “yellow zone” treatment when the PEF % pred decreases during long-term PEF monitoring to prevent acute exacerbations of asthma. The most common trigger for acute exacerbations of asthma is upper respiratory tract infections. Acute exacerbations due to air pollution have significantly higher variations in PEF% pred than other triggers.

## Data Availability

All data generated or analysed during this study are included in this published article.

## Declarations

### Author’s information

Xiongbin Chen and Peng Han are the first author of this paper and Kunling Shen is corresponding author.

## Abbreviations

PEF: peak expiratory flow
CCAAP: China Children’s Asthma Action Plan
FEV_1_: forced expiratory volume in one second
